# Supplementary material for: Resolving the full spectrum of human genome variation using Linked-Reads
Source: Genome Res. 2019 Apr;29(4):635–45. doi: 10.1101/gr.234443.118 (PMC6442396; doi:10.1101/gr.234443.118)
Supplement: Supplemental Material [file supp_gr.234443.118_Supplemental_longranger-2.2.zip › longranger-2.2.tar.bz2!/lr2.2-public/lib/stan/cmdstan/stan/doc/api/html/math_2functions_2log1p__exp_8hpp_source.html]

Stan: src/stan/math/functions/log1p\_exp.hpp Source File


|  |  |
| --- | --- |
|  | Stan  2.5.0  probability, sampling & optimization |


- Main Page
- Related Pages
- Namespaces
- Classes
- Files

- File List
- File Members

All Classes Namespaces Files Functions Variables Typedefs Enumerations Enumerator Friends Macros Pages

- src
- stan
- math
- functions

log1p\_exp.hpp

Go to the documentation of this file.

1 #ifndef STAN\_\_MATH\_\_FUNCTIONS\_\_LOG1P\_EXP\_HPP

2 #define STAN\_\_MATH\_\_FUNCTIONS\_\_LOG1P\_EXP\_HPP

3

4 #include <boost/math/tools/promotion.hpp>

5 #include <stan/math/functions/log1p.hpp>

6

7 namespace stan {

8  namespace math {

9

42  template <typename T>

43  inline typename boost::math::tools::promote\_args<T>::type

44  log1p\_exp(const T a) {

45  using std::exp;

46  // like log\_sum\_exp below with b=0.0; prevents underflow

47  if (a > 0.0)

48  return a + log1p(exp(-a));

49  return log1p(exp(a));

50  }

51

52  }

53 }

54

55 #endif

stan::math::log1p\_exp

boost::math::tools::promote\_args< T >::type log1p\_exp(const T a)

Calculates the log of 1 plus the exponential of the specified value without overflow.

**Definition:** log1p\_exp.hpp:44

stan::math::log1p

boost::math::tools::promote\_args< T >::type log1p(const T x)

Return the natural logarithm of one plus the specified value.

**Definition:** log1p.hpp:39

stan::math::exp

Eigen::Matrix< T, Rows, Cols > exp(const Eigen::Matrix< T, Rows, Cols > &m)

Return the element-wise exponentiation of the matrix or vector.

**Definition:** exp.hpp:18

log1p.hpp

stan::agrad::exp

fvar< T > exp(const fvar< T > &x)

**Definition:** exp.hpp:16


---

[ Stan Home Page ]

*© 2011–2014,
Stan Development Team.*
